# Antibiotic therapy in viral airway infections (ATHENIAN): study protocol for an open labeled randomized controlled pragmatic trial to evaluate the efficacy and safety of discontinuing antibiotic therapy in adult patients infected with respiratory viruses

**DOI:** 10.1186/s13063-026-09730-3

**Published:** 2026-04-21

**Authors:** Magrit Jarlsdatter Hovind, Jan Erik Berdal, Jūratė Šaltytė Benth, Olav Dalgard, Magnus Nakrem Lyngbakken

**Affiliations:** 1 Department of Infectious Diseases, Division of Medicine, Akershus University Hospital, Sykehusveien 25, Lørenskog, 1478 Norway; 2 Institute of Clinical Medicine, University of Oslo, Oslo, Norway; 3 Health Services Research Unit, Akershus University Hospital, Sykehusveien 25, Lørenskog, 1478 Norway

**Keywords:** Respiratory tract infections, Influenza, Respiratory Syncytial Virus, Human Metapneumovirus, Parainfluenza virus, Antibiotic therapy, Pragmatic clinical trial

## Abstract

**Background:**

Reducing inappropriate use of antibiotics is essential to combat antimicrobial resistance. Antibiotics are widely prescribed upon hospital admission for acute respiratory infections, but it remains unknown whether it is safe to discontinue antibiotics when a respiratory virus is detected in these patients. With this randomized trial, we aim to assess the efficacy and safety of discontinuing antibiotic therapy in patients admitted to the hospital with a polymerase chain reaction test positive for respiratory virus and no clear evidence of bacterial infection.

**Methods:**

ATHENIAN is an ongoing multicenter, open-label, pragmatic, randomized non-inferiority trial. We aim to recruit 400 adult patients initiated on antibiotic therapy at admittance to one of the 10 study sites in Norway and with a positive polymerase chain reaction test for influenza virus, human metapneumovirus, respiratory syncytial virus, or parainfluenza virus. The participants are randomized to intervention, discontinuation of antibiotic therapy, or control, continuation of antibiotic therapy at the discretion of the treating physician. We hypothesize that discontinuation of antibiotic therapy is safe and non-inferior to continuation of antibiotic therapy. The primary outcome, assessed at 120 h after randomization, is early clinical response, defined as survival with symptom improvement without receipt of rescue antibacterial therapy. Secondary outcomes include all-cause in-hospital and 30-day mortality, duration of hospital admission, days of therapy with antibiotics, rescue antibiotic therapy during hospital admission, new antibiotic therapy for presumed airway infection up to 30 days after hospital discharge, and hospital readmissions within 30 days after hospital discharge.

**Discussion:**

To our knowledge, ATHENIAN is the first randomized controlled trial assessing the safety and efficacy of antimicrobial de-escalation based on multiplex nucleic acid amplification test results. Addressing this knowledge gap using a pragmatic study design will provide valuable insights that may influence treatment algorithms and antibiotic prescription practices. The study has the potential to contribute to a reduction in the inappropriate use of antibiotics and the emergence of antimicrobial resistance.

**Trial registration:**

ClinicalTrials.gov NCT05045612. Registered on September 7, 2021.

**Supplementary Information:**

The online version contains supplementary material available at 10.1186/s13063-026-09730-3.

## Structured summary {1b}

**Table:** 

Item	Description
Primary Registry and Trial Identifying Number {4}	ClinicalTrials.gov, NCT05045612. Registered on September 7, 2021. https://clinicaltrials.gov/study/NCT05045612
Secondary Identifying Numbers	EU CT#2023–509286-20-00
Source(s) of Monetary or Material Support	The trial received funding from the National Program for Clinical Therapy Research in the Specialist Health Services (KLINBEFORSK) in December 2023. The funder has no role in design of the study, data collection, analysis, interpretation, or preparation of this manuscript.
Primary Sponsor and contact information {3b}	Jan Erik Berdal, Department of Infectious Diseases, Akershus University Hospital, 1478 Lørenskog, Norway. E-mail: jan-erik.berdal@ahus.no.
Role of sponsor and funder {3c}	The sponsor is Akershus University Hospital, represented by Jan Erik Berdal, Head of the Department of Infectious Diseases. He is a member of the ATHENIAN trial steering committee, and was involved in study design, data collection, and preparation of this manuscript.
Contact for Public Queries	https://www.ahus.no/kliniske-studier/athenian-antibiotikabehandling-ved-virale-luftveisinfeksjoner/ Magnus Nakrem Lyngbakken, principal investigator, m.n.lyngbakken@medisin.uio.no
Contact for Scientific Queries	Magnus Nakrem Lyngbakken, principal investigator, m.n.lyngbakken@medisin.uio.no
Public Title	Antibiotic therapy in viral airway infections
Scientific title	Antibiotic therapy in viral airway infections (ATHENIAN): study protocol for an open labeled randomized controlled pragmatic trial to evaluate the efficacy and safety of discontinuing antibiotic therapy in adult patients infected with respiratory viruses
Countries of Recruitment	Norway
Health Condition(s) or Problem(s) Studied	Viral airway infection
Intervention(s)	Discontinuation of antibiotic therapy
Key Inclusion and Exclusion Criteria	Inclusion criteria: Hospitalized, Adults 18 years or older, A nasopharyngeal swab positive for influenza virus, parainfluenza virus, respiratory syncytial virus or human metapneumovirus, CRB65 ≤ 2 at time of inclusion1, On antibiotic therapy as instituted by the receiving physician from the emergency department, Signed informed consent Exclusion criteria: Requiring admission to intensive care unit at screening, Requiring high-flow oxygen therapy or non-invasive ventilation at screening, Signs of severe pneumonia, Not immunocompetent, Severe acute respiratory syndrome coronavirus 2 positive, Bacteremia, Urine antigen test positive for legionella, Any other infection necessitating antibiotic treatment, Antibiotic use for assumed airway infection within the last 24 h before admission to hospital, Time from initiation of antibiotic therapy to screening > 48 h
Study Type	Two-arm, open labeled, multicenter randomized controlled pragmatic non-inferiority trial
Date of First Enrollment	January 13, 2022
Sample Size	400
Primary outcome(s)	Early clinical response assessed at 120 h after randomization, defined as survival with symptom improvement without receipt of rescue antibacterial therapy
Key Secondary outcome(s)	In-hospital mortality, Mortality at 30 days, Duration of hospital admission, Days of therapy with antibiotics, Rescue antibiotic therapy during hospital admission, New antibiotic therapy for presumed airway infection up to 30 days after discharge, Hospital readmissions up to 30 days after discharge
Ethics Review	The Regional Committee for Medical Research Ethics, Norway, REC#213847. The Norwegian Medical Products Agency, EudraCT#2021–004248-11/EU CT#2023–509286-20-00.
Individual Trial Participant Data sharing statement	Individual trial participant data will not be shared.

### Protocol version {2}

Version 1.14, dated March 18, 2025.

## Introduction

### Background and rationale {9a}

Antimicrobial resistance (AMR) has been declared one of the greatest threats to global health with an estimated 4.95 million associated deaths yearly [1, 2]. Inappropriate use of antibiotics, a main driver of AMR [3], is common in respiratory infections in adults [4], as the causative agent frequently is viral [5]. Acute respiratory infections are prevalent [6] and overtreatment with antibiotics makes it a possible goal for antibiotic stewardship efforts. Polymerase chain reaction (PCR) tests, including point-of-care testing, have improved diagnostics in acute respiratory infections and enable the detection of multiple viruses within a short time after hospitalization [7]. Although the utilization of PCR increases the diagnostic yield, previous studies have demonstrated a variable effect on antibiotic prescription practices [8–10], possibly due to the difficulty of ruling out bacterial-viral co-infections. Risk of bacterial-viral co-infection exists, but it remains uncertain how frequent this occurs and the consequences of discontinuing antibiotics in patients hospitalized with acute respiratory infections, a positive PCR test for respiratory viruses, and without clear signs of a bacterial infection are not known. In this context, we conduct a pragmatic randomized trial in which patients with a positive PCR for a respiratory virus are randomized to intervention, discontinuation of antibiotic therapy, or control, continuation of antibiotic therapy. The aim is to provide evidence that may improve prescription practices and ultimately reduce the usage of futile antibiotic therapy.

### Explanation for the choice of comparator {9b}

The standard of care is chosen as the comparator as the objective is not to compare different antibiotics. Patients in the comparator arm will be administered antibiotics at the doctor’s discretion. In Norway, intravenous benzylpenicillin is the recommended treatment of moderately severe community-acquired pneumonia requiring hospitalization [13].

### Objectives {10}

The overall objective of this trial is to assess whether discontinuation of antibiotic therapy is safe and non-inferior to continuation of antibiotic therapy in patients with a positive PCR for respiratory viruses. Specifically, we aim to assess the impact of discontinuing antibiotic therapy on early clinical response, in-hospital and 30-day mortality, duration of hospital admission, days of therapy with antibiotics, rescue antibiotic therapy during admission, and new prescriptions of antibiotics and hospital readmissions up to 30 days after hospital discharge.

## Methods: patient and public involvement, and trial design

### Patient and public involvement {11}

A cooperation with representatives from two patient organizations has been established to ensure patient and public involvement throughout study conduct.

### Trial design {12}

**Fig. 1:**
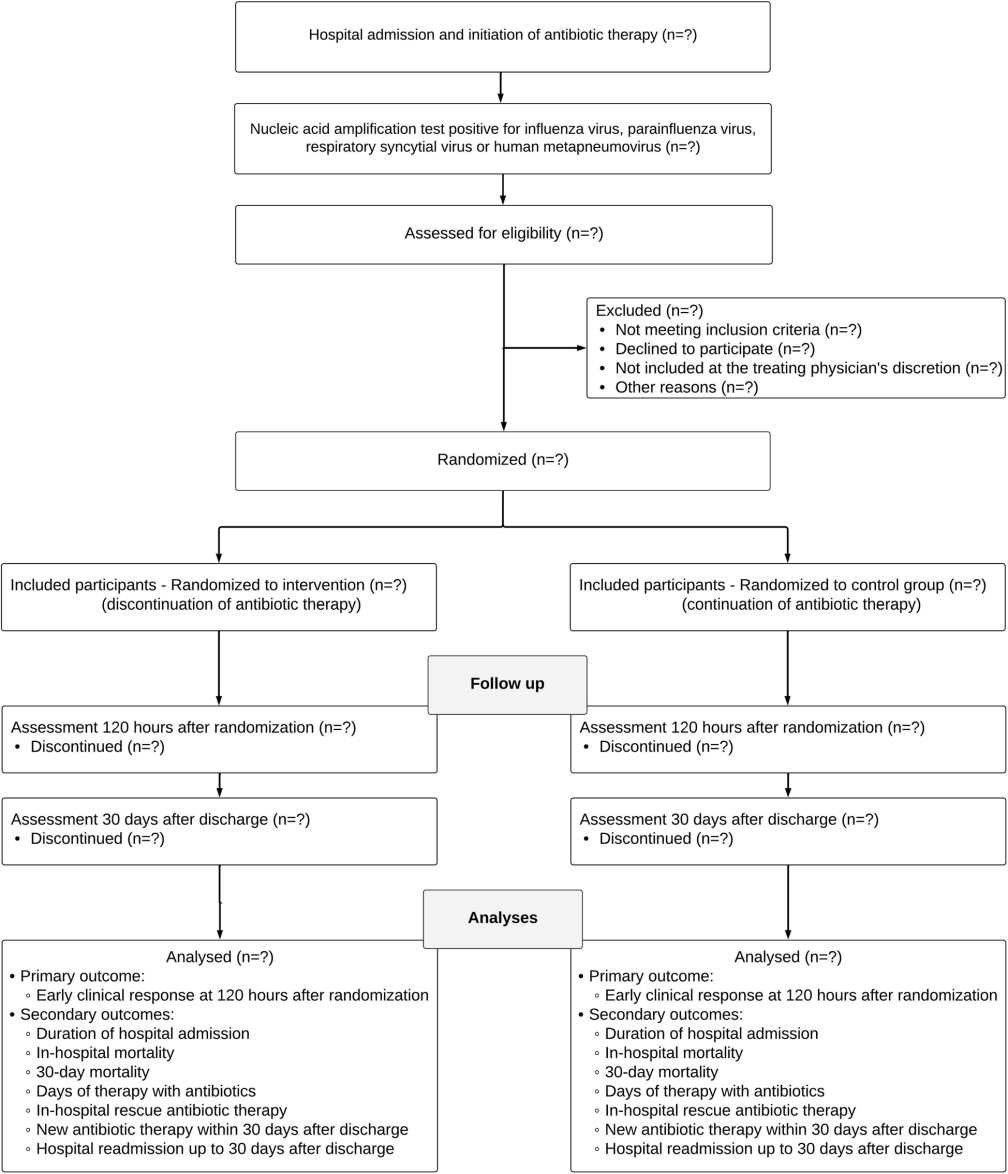
Flow diagram of the inclusion of study participants, randomization, assessments, and outcomes in the ATHENIAN trial

ATHENIAN is an investigator-initiated, open-label, two-arm, randomized controlled non-inferiority trial (Fig. 1). The trial is a pragmatic trial [11] conducted in a real-world emergency setting with a relatively unselected and clinically relevant patient population. Some exclusion criteria apply mainly for safety reasons. Other pragmatic features of the trial include limited use of resources beyond usual clinical care, few study-specific procedures, endpoints that are meaningful and relevant to the patients, use of standard of care as the comparator without blinding or placebo, and collection of data readily available in the electronic medical record (EMR).

## Methods: participants, interventions, and outcomes

### Trial setting {13}

The trial commenced as a single-center study at Akershus University Hospital (AHUS) in January 2021 and was extended to a multicenter study from January 2024. The following 10 hospitals have started patient recruitment: AHUS, Oslo University Hospital, Stavanger University Hospital, Vestfold Hospital Trust, Drammen Hospital, Østfold Hospital, Sørlandet Hospital Kristiansand, University Hospital of North Norway, Telemark Hospital and Bærum Hospital. These 10 hospitals are chosen based on interest of participation, geographical variation, and size of patient population, and cover a population of approximately 2.5 million. All sites have a dedicated study team led by a principal investigator with experience in infectious diseases and/or clinical research.

#### Characteristics of the people who are needed for the trial

**Table:** 

Characteristic	The people we would expect to see included
Age	Median 70 years old. 65% of patient population ≥65 years old.
Sex	50% males, 50% females.
Gender	
Race, ethnicity and ancestry	Data not collected.
Socioeconomic status	Data not collected.
Geographic location	All patients are recruited from primary, secondary or tertiary hospitals throughout Norway, covering all health regions.
Other characteristics relevant to the trial	

### Eligibility criteria for participants {14a}

Patients admitted to the hospital with symptoms or signs of an acute respiratory infection are routinely examined with a nasopharyngeal swab and a subsequent specific PCR test for respiratory viruses. To identify potential eligible patients, the study staff at each site receive laboratory reports of all positive PCR tests from the Department of Microbiology with a maximum 24-h interval. The inclusion and exclusion criteria are presented in Table 1.

**Table 1:** Inclusion and exclusion criteria

Inclusion criteria	Exclusion criteria
• Hospitalized • Adults 18 years or older • A nasopharyngeal swab positive for influenza virus, parainfluenza virus, respiratory syncytial virus or human metapneumovirus • CRB65 ≤ 2 at time of inclusion^a^ • On antibiotic therapy as instituted by the receiving physician from the emergency department • Signed informed consent	• Requiring admission to intensive care unit at screening • Requiring high-flow oxygen therapy or non-invasive ventilation at screening • Signs of severe pneumonia (abscesses, massive pleural effusion, a well-defined lobar infiltrate on chest X-ray strongly suggestive of bacterial etiology) • Not immunocompetent (i.e., on active chemotherapy, corticosteroid therapy equaling ≥ 20 mg prednisolone daily for ≥ 4 weeks, chronic immunosuppression due to solid organ transplant) • Severe acute respiratory syndrome coronavirus 2 positive • Bacteremia • Urine antigen test positive for legionella • Any other infection necessitating antibiotic treatment • Antibiotic use for assumed airway infection within the last 24 h before admission to hospital • Time from initiation of antibiotic therapy to screening > 48 h

^a^CRB-65 is a severity score for community-acquired pneumonia, used to predict 30-day mortality risk. The score is based on four clinical components, each of which gives one point: C - new confusion, R - respiratory rate ≥ 30/min, B - hypotension (systolic ≤ 90 mm Hg or diastolic ≤ 60 mm Hg), and 65 - age ≥ 65 years. Score 0: Low risk. Score 1–2: Moderate risk. Score ≥3: High risk [12]

### Eligibility criteria for sites and those delivering interventions {14b}

Eligible sites are primary, secondary and tertiary care hospitals with an emergency department, PCR testing facilities and study staff available for screening and inclusion of patients. Interventions will be delivered by consultants and nurses authorized by the principal investigator after completing Good Clinical Practice training and protocol training.

### Who will take informed consent? {32a}

Following the eligibility check, the investigator approaches the patient in the ward to collect informed consent. The patients receive verbal and written information about the study including the rationale of the study, data protection rights, study-specific procedures, possible risks and benefits of participation, and the right to withdraw the consent. The investigator ensures that the patient comprehends the information provided and that consent is given voluntarily, and excludes patients in case of confusion, a language barrier or other factors affecting the consent process or the collection of endpoint data.

### Additional consent provisions for collection and use of participant data and biological specimens {32b}

Not applicable as no biological specimens are collected in this study.

## Intervention and comparator

### Intervention and comparator description {15a}

Following randomization to the intervention group, the investigators discontinue antibiotics immediately in the electronic medical chart while all other treatments follow clinical routine. Patients randomized to the control group will receive the standard of care at the treating physician’s discretion. Patients in both groups are discharged at the discretion of the treating physicians.

### Criteria for discontinuing or modifying allocated interventions {15b}

Withdrawal of the informed consent and incorrect enrolment are the only reasons for discontinuing allocated interventions. Antibiotics may be reinstated by the treating physician if deemed necessary without consulting or notifying the study team. In order not to cause increased antibiotic usage, the trial permits discontinuation of antibiotics in the control group if the treating physician decides this to be appropriate. Data on reinstatement of antibiotic therapy is collected as part of the primary outcome and as a secondary outcome. Data on total days of therapy with antibiotics will give indications on the discontinuation of antibiotic therapy in the control group.

### Strategies to improve adherence to interventions/comparators {15c}

As this is a pragmatic trial mimicking real-life experience, there are no strategies to improve adherence to the intervention except ensuring that the patient and the treating physician are well informed about the trial.

### Concomitant care permitted or prohibited during the trial {15d}

After randomization, treatment in both groups follows clinical routine, and all concomitant care and medications are accepted. As this is a stop-study, possible interactions with antibiotic therapy are not relevant.

### Ancillary and post-trial care {34}

The endpoint assessment 120 h after randomization is performed by phone if the patient is discharged. Except for this study-specific procedure, follow-up is limited to clinical routine which usually does not include any follow-up after discharge of patients with acute respiratory infection. Participants are informed to visit a doctor if there is worsening of symptoms or other signs of deterioration after discharge to ensure that antibiotics will be reinstated if needed. No compensation will be given to those in need of new antibiotic therapy or readmissions.

### Outcomes {16}

In line with the pragmatic design, the outcomes of this trial are relevant and important to the patient. The primary outcome is early clinical response, assessed at 120 h after randomization. Early clinical response is a composite endpoint combining survival, receipt of rescue antibacterial therapy and symptom improvement. Symptoms are measured by a symptom severity score consisting of four elements (cough, pleuritic chest pain, shortness of breath and sputum) each scored on a four-point scale (absent, mild, moderate, or severe) (Table 2). We acknowledge that a self-reported symptom score is prone to variability and inconsistency and may not accurately reflect the clinical picture. However, symptom improvement is an important endpoint from the patient’s perspective, and this symptom severity score is recommended by the US Food and Drug Administration [14] and used as an outcome in prior studies of treatment effect in community-acquired pneumonia [15]. As mortality in this group of intermediate-risk patients is relatively low, mortality as an endpoint would require a considerably higher number of participants.

**Table 2:** Symptom severity assessment^a^ performed at inclusion and 120 h after randomization

Absent	Mild	Moderate	Severe
Cough?			
No cough or resolution of cough to pre-CAP baseline	Cough present but it does not interfere with subject’s usual daily activities	Cough present, frequent and it does interfere with some of the subject’s usual daily activities	Cough is present throughout the day and night; it limits most of the subjects’ usual daily activities and sleep patterns
Pleuritic chest pain?			
No chest pain or resolution of chest pain to pre-CAP baseline	Chest pain present occasionally with deep breathing but it does not interfere with subject’s usual daily activities	Chest pain is present with normal breath and it does interfere with the subject’s usual daily activities	Chest pain is present at rest and/or with shallow breathing; it limits most of the subject’s usual daily activities
Shortness of breath?			
No shortness of breath or resolution of shortness of breath to pre-CAP baseline	Shortness of breath with strenuous activities only but it does not interfere with subject’s usual daily activities	Shortness of breath with usual activities and it does interfere with the subject’s usual daily activities	Shortness of breath with minimal exertion or at rest; it limits most of the subject’s usual daily activities
Phlegm/sputum production?			
No coughing up of phlegm/sputum or resolution of coughing up phlegm/sputum to pre-CAP baseline	Subject coughs up a small amount of phlegm/sputum	Subject coughs up a moderate amount of phlegm/sputum	Subject coughs up a large amount of phlegm/sputum

^a^Symptom improvement is defined as improvement of one or more levels relative to baseline in two or more symptoms and no worsening of one or more levels in other symptoms

From Stets et al. N Engl J Med. 2019; 380:517–527 [15]

The following secondary endpoints will be assessed:

- In-hospital mortality
- Mortality at 30 days
- Duration of hospital admission
- Days of therapy with antibiotics
- Rescue antibiotic therapy during hospital admission
- New antibiotic therapy for presumed airway infection up to 30 days after discharge
- Hospital readmissions up to 30 days after discharge

### Harms {17}

All antibiotic agents used in the study are approved by the Norwegian Medical Agency and have established side effect profiles. Additionally, this trial is designed as a stop-study where the intervention is discontinuation of antibiotics. Consequently, the frequency of regular side effects such as nausea and rash are not relevant and not reported as adverse events. Participation in the study carries a risk for those randomized to the intervention group as a bacterial infection cannot be ruled out and undertreatment of a potential bacterial co-infection is possible. The participants receive information about the risk of harm at enrolment. The trial steering committee considers this risk to be low with the precautions taken in the study such as inclusion of only low-to-moderate-risk patients, observational time with antibiotics before inclusion and allowance of rescue antibiotic therapy at any time. We collect data on the need for rescue antibiotics during hospitalization and new antibiotic therapy within 30 days after discharge. Complications or clinical deterioration, meeting the definition of serious adverse events (SAE), occurring between randomization and the follow-up assessments, are reported for both the intervention group and the control group. The SAEs are reported to the sponsor within 24 h via standardized forms in Viedoc, and the relation to the intervention is assessed by the medical monitor. The numbers and proportions of participants experiencing SAEs will be published.

### Participant timeline {18}

The time schedule of trial activities is presented in Table 3.

**Table 3:** Schedule of trial activities in ATHENIAN

	STUDY PERIOD							
	Admission	Enrolment	After randomization					Close-out
TIMEPOINT		0	*24 h*	*48 h*	*72 h*	*96 h*	*120 h*	*30 days after discharge*
Nasopharyngeal test	X							
Eligibility screen		X						
Informed consent		X						
INTERVENTIONS:								
Discontinuation of antibiotic therapy (intervention)		X						
Continuation (Control)								
ASSESSMENTS:								
Record of concurrent medication	X^a^							
Physical examination	X^a^		X^a^	X^a^	X^a^	X^a^	X^a^	
Blood samples	X^a^		X^a^	X^a^	X^a^	X^a^	X^a^	
Symptom severity assessment		X					X^b^	
Collection of baseline data		X						
SAE and SUSAR			X^c^	X^c^	X^c^	X^c^	X^c^	
Mortality			X	X	X	X	X	X
New antibiotic therapy							X	X
Readmission							X	X
Data extraction^d^		X					X	X

*Abbreviations*: *SAE *Serious Adverse Events, *SUSAR* Suspected Unexpected Serious Adverse Reaction

^a^At the discretion of the admitting/treating physician

^b^By phone if discharged

^c^If discharged before the end of treatment, assessment of SAE is performed by phone directly to the patient at 120 h

^d^Data extracted include radiology reports, biochemistry, microbiology, and antibiotic therapy and antiviral therapy prescribed during admission

### Sample size {19}

Power analyses were performed in STATA in cooperation with an independent statistician at AHUS. We calculated the sample size based on assumptions about the proportion of study patients with early clinical improvement from randomization to 120 h. Patients with influenza usually improve after 3–7 days [16] and we assumed an early clinical improvement of 90% both in the intervention group and the control group. To demonstrate non-inferiority of the intervention with a non-inferiority margin of 10%, one-sided significance level of 2.5% and 90% power, we are required to include a total of 380 patients. We aim to include 400 patients with 200 patients in each study arm to account for incorrect inclusions, withdrawal of consent and loss to follow-up. We did not adjust the power calculations for potential cluster effect due to the multicenter nature of the study, as the treatment and outcomes are expected to be similar across all study sites.

### Recruitment {20}

Recruitment is performed at all sites based on electronic surveillance of positive airway samples for respiratory viruses. Patients are recruited from all hospital departments, typically, but not exclusively, from departments of internal medicine. Due to respiratory virus seasonality, patient screening is not performed in the summer season, and whether screening is performed outside regular working hours varies from site to site depending on capacity and availability of the study team. In the initial phase of the study, fewer patients than expected were recruited. With efforts such as expansion into a multicenter study, dedicated study teams at each site, close follow-up by the coordinating center, and agreements including the number of expected inclusions, and with an estimated enrollment rate of 80–90 patients per year, we expect to include the target number of patients within a reasonable time.

## Assignment of interventions: randomization

### Sequence generation: who will generate the sequence {21a}

The allocation sequence was prepared by an independent statistician.

### Sequence generation: type of randomization {21b}

Eligible patients are allocated in a 1:1 ratio to the intervention group or the control group, using a computer-generated block-randomization procedure. Separate randomization sequences have been prepared for each site to assure balanced arms within the site.

### Allocation concealment mechanism {22}

The allocation sequence has been imported into the clinical data management system, Viedoc. Upon enrollment of a new patient, the randomization allocation appears automatically for the investigator who documents the randomization allocation in the EMR and informs the treating physician and the patient.

### Implementation {23}

The allocation sequence and block sizes are not accessible to patients or investigators enrolling patients, thereby preventing selection bias.

## Assignment of interventions: blinding

### Who will be blinded {24a}

In accordance with the pragmatic study design, neither patients nor investigators are blinded. The study statistician will be blinded to the group allocation until all analyses will be performed.

### How will blinding be achieved {24b}

Not applicable as ATHENIAN is an open-label trial.

### Procedure for unblinding if needed {24c}

Not applicable as ATHENIAN is an open-label trial.

## Data collection and management

### Plans for assessment and collection of outcomes {25a}

All data are primarily collected as part of clinical routine, and the data extraction is limited to data necessary to address the hypothesis and aims of the study. In case of missing baseline data, the investigators collect these data directly from the patient at inclusion. Baseline data (comorbidities, level of dependency, smoking status, symptoms and symptom onset, and vital signs), laboratory results, microbiological findings, radiological reports, and data on antibiotic and antiviral treatment are retrieved from the EMR. For assessment of the primary endpoint the symptom severity score is collected at inclusion and after 120 h, either by phone or in the ward if still hospitalized. Data collected from the EMR at follow-up after 120 h and 30 days after discharge include laboratory results, microbiological results, mortality, and readmissions. Additionally, data on new prescriptions of antibiotic therapy is collected from the national digital prescription system. Data is entered into the clinical data management system, Viedoc, by investigators at each site, using a unique study ID generated at randomization for each participant.

### Plans to promote participant retention and complete follow-up {25b}

As a pragmatic study, this trial involves few study-specific procedures and assessments, and minimal effort is required from the patient to participate. To assure the collection of symptom severity score at 120 days after randomization, the participants are informed about the importance of the collection of endpoint data, and they are encouraged to answer their phones when contacted. No effort is required from the participant to complete follow-up 30 days post-discharge as all data are available from the EMR and national digital prescription system.

### Data management {26}

The unique study ID is used for all data management, and the code list linking the study ID and the social security number of the patient is stored in the Investigator’s Site File locally at each site. Investigators at each site manually enter all data into Viedoc. To minimize missing data, investigators are required to report the reason for missing data when completing the case report form. All data will be kept in encrypted secure areas and archived for 25 years after finalization of the study according to international regulations.

### Confidentiality {33}

All data will be handled in accordance with the data protection legislation. Only the local study team have access to the code list of patients included at their site. Thus, all data is anonymized before extraction from Viedoc for analysis at the coordinator center. The results will be presented on group level without identifying individual participants.

## Statistical methods

### Statistical methods for primary and secondary outcomes {27a}

The planned statistical analyses are described in the statistical analysis plan V1.5 (supplementary material). All statistics will be presented by treatment group. The analyses of the primary endpoint will be performed on the Full Analysis Set (FAS), corresponding to the modified intention-to-treat (mITT) population, and the per protocol (PP) population. The primary endpoint will be analyzed as the difference in proportions between the intervention and control groups (intervention–control). The confidence interval (CI) for the difference will be calculated using the standard normal approximation (Wald method). A one-sided 97.5% CI will be presented. Non-inferiority will be concluded if the lower bound of the 97.5% CI exceeds the prespecified non-inferiority margin of 10%. As a sensitivity analysis, the effect of the intervention on a version of the primary endpoint where criterion 3 (i.e., no rescue antibiotic therapy) is removed will be assessed using the same non-inferiority margin. The analyses of secondary endpoints will be performed on the intention-to-treat (ITT) population and the PP population using *χ*^2^-test, Independent-samples *t*-test or non-parametric alternatives, the Kaplan-Meier method and log-rank test. All randomized patients with any data on safety (Safety Analysis Set (SAS)) will be included in the safety analysis where numbers and proportions of participants experiencing SAEs and suspected unexpected serious adverse reactions (SUSARS) will be presented. There will be no adjustment for baseline or other covariates.

### Who will be included in each analysis {27b}

The FAS (corresponding to mITT) will include all randomized patients with one baseline and one post-randomization evaluation of the primary endpoint. The ITT population will include all randomized participants, analyzed according to their assigned treatment groups regardless of treatment adherence, protocol deviations, or availability of post‑baseline data. The PP population will include all randomized patients without major protocol violations. The SAS includes all subjects with any safety information after baseline.

### How missing data will be handled in the analysis {27c}

The primary analysis will be performed both on the mITT population and the PP population. If the amount of missing primary endpoint data is substantial and considered likely to meaningfully influence the trial conclusions, a sensitivity analysis will be conducted using inverse probability weighting, where weights are derived from relevant patient characteristics to account for differential missingness and maintain balance between treatment groups. The same consideration will apply for secondary endpoints.

### Methods for additional analyses (e.g. subgroup analyses) {27d}

Primary and secondary analyses will be performed separately on the subgroups defined by sex, age above/below 65 years, C-reactive protein above/below 100 mg/L, type of virus detected, patients with and without radiographic evidence of pneumonia and patients with and without specific comorbidities (diabetes mellitus, obstructive pulmonary disease, cardiovascular disease).

### Interim analyses {28b}

Interim analyses, conducted to oversee the risk–benefit balance and enable early termination both for benefit and futility, are planned after 40 (performed May 2023), 100 (performed October 2025) and 200 completed patients (planned Spring 2026). Additionally, extraordinary interim analyses may be conducted if deemed necessary by the sponsor.

### Protocol and statistical analysis plan {5}

The full study protocol and statistical analysis plan are available on ClinicalTrials.gov. A de-identified dataset and statistical code may be made available on reasonable request.

## Oversight and monitoring

### Composition of the coordinating center and trial steering committee {3d}

This is an investigator-initiated trial commenced by a research group at the coordinating center, AHUS. The research group, including the national coordinating investigator and other investigators with broad experience on infectious diseases and/or conduction of clinical trials, manages the trial together with one investigator from each site through the trial steering committee. Regular meetings are held during the study to discuss the conduction of the trial, protocol adherence, and safety. The research group at the coordinating center provides ongoing support to investigators and follows up with all study sites before and after each winter season, as well as in the event of adverse events, protocol deviations or other challenges related to study conduction. Newsletters are also sent out to all study staff frequently, with important information and updates on patient recruitment for encouragement.

### Composition of the data monitoring committee, its role and reporting structure {28a}

The data monitoring committee (DMC) consists of two clinicians with experience from infectious diseases/virology and clinical studies, and one independent statistician. The members are employed at other hospitals or departments and are independent of the sponsor. They were not involved in study design and do not take part in the study as investigators. In accordance with the DMC charter, they overview the safety and efficacy of the intervention with meetings after 40, 100, and 200 completed patients. The advisory recommendations of the DMC are reported to the trial steering committee after each meeting and may include stopping the trial if there is a safety concern which warrants stopping the trial, if the *p*-value of the no treatment effect hypothesis test is below the threshold specified in the DMC charter or if there is insufficient recruitment. The trial steering committee is only informed of whether the DMC recommends continuation or discontinuation of the study, not the results of the interim analyses. Continuation of the study was recommended following the first two DMC meetings, held in May 2023 and October 2025.

### Frequency and plans for auditing trial conduct {29}

The trial monitoring is coordinated by the Clinical Trial Unit at Oslo University Hospital and performed in accordance with the monitor plan developed based on the risk assessment. The monitor visits each site at initiation, after 10 patients, and at least once a year, and monitors study training, documentation of source data, collection of endpoints, reporting of SAE, protocol deviations, and the informed consent process. Additionally, as outlined, the DMC is overviewing the safety and the efficacy of the intervention.

### Protocol amendments {31}

New protocol versions are submitted for approval to the Regional Committees for Medical and Health Research and the Norwegian Medical Products Agency when required. Once approved, new protocol versions are stored in the Investigator’s Site File and the Trial Master File in Viedoc. Protocol amendments are communicated to the Principal Investigator and the study teams at each site. The Principal Investigator is required to sign all new versions and document training of study staff if substantial protocol amendments are made. Protocol deviations are reported via standardized forms in Viedoc and followed up by the national coordinating Investigator.

### Dissemination policy {8}

The results of this study will be published in peer-reviewed medical journals and in national and international media. Furthermore, we will present the results at national and international scientific conferences, and to important organizations and agencies involved in antibiotic stewardship.

## Discussion

To our knowledge, the ATHENIAN trial is the first randomized clinical trial designed to assess whether physicians should continue or discontinue antibiotics following a positive airway sample for respiratory viruses in patients hospitalized with acute respiratory infection. The risk of bacterial-viral co-infection and the need for antibiotic therapy in these patients are debated topics with conflicting evidence. Investigating the prevalence of such co-infection is outside the scope of this study and would require different methodology. While we acknowledge that a viral-bacterial interplay is biologically plausible, our non-inferiority study aims to address the central clinical question of whether stopping antibiotic treatment has meaningful negative clinical effects, and whether it is safe to discontinue antibiotics in these patients in the absence of definite evidence of bacterial infection by the usual diagnostic methods used in emergency departments such as chest X-ray and blood cultures. We acknowledge that some patients may benefit from antibiotic therapy. However, the risk of severe outcome is low, as antibiotics can be reintroduced at any time. The societal interests with regard to increasing AMR outweigh the individual benefit of possible symptom relief and the individual risk of a prolonged disease course.

Evaluating this research question using a pragmatic study design with a relatively unselected patient population, meaningful endpoints, few study-specific procedures, and few additional resources beyond what is already available in routine clinical practice ensures the feasibility of the study, provides data with increased applicability and generalizability, and better addresses this real-world clinical dilemma than a more traditional study design.

The trial has certain limitations and has encountered challenges regarding recruitment during the initial phase. After two winter seasons as a single-center study with insufficient enrollment, the necessity of expanding to a multicenter trial became evident. As the intervention is the discontinuation of antibiotics, a requirement for participation is treatment with antibiotic therapy. During the first phase of the study, we experienced that fewer patients than expected were treated with antibiotics, possibly due to changes in prescription practices after the Covid-19 pandemic and/or the rapid turnaround time for PCR tests. However, approximately 50% of patients receive antibiotics at prescreening which highlights the unmet need of this trial. Although the exclusion criteria are few to include an unselected patient population, a high proportion of patients assessed for eligibility fulfill one or more exclusion criteria. Among the most common reasons for exclusion are prehospital antibiotic usage, admission to the intensive care unit, treatment with non-invasive ventilation, immunosuppression and lack of ability to consent. Particularly, the exclusion of the latter group reduces the generalizability and applicability of the results, but it would not be ethical to conduct the study including patients who do not comprehend the risk of participation. Another limitation is reluctance from the treating physician to include patients in the study, despite the fact that the patient does not exhibit any exclusion criteria precluding participation. However, this pertains so far to only a very minor proportion of patients and the great majority of physicians agree on study inclusion. In some cases of particularly fragile patients, despite being eligible, study inclusion has not been possible due to hesitancy from the treating physician.

In conclusion, despite certain limitations and challenges, a pragmatic randomized trial assessing the safety and efficacy of discontinuing antibiotics following detection of a respiratory virus will provide direly needed clinical evidence. The trial has the potential to influence treatment algorithms of viral lower airway infections, possibly leading to a reduction in antibiotic usage crucial to combat antimicrobial resistance.

## Trial status

Current protocol version: V1.14, dated 18 March 2025. The study was registered at ClinicalTrials.gov (NCT05045612) on September 7, 2021, before patient recruitment commenced at AHUS in January 2022. Patient recruitment as a multicenter study at other sites started in December 2023. Per March 19, 2026, 248 patients have been included and we expect to be recruiting patients for the following 2 years.

## Supplementary Information

Supplementary Material 1.

## Data Availability

The final dataset will be available on reasonable request when the study is completed.

## Abbreviations

AHUS: Akershus University Hospital
AMR: Antimicrobial resistance
DMC: Data Monitoring Committee
EMR: Electronic medical record
FAS: Full Analysis Set
ITT: Intention to treat
mITT: Modified intention to treat
PCR: Polymerase chain reaction
PP: Per protocol
SAE: Serious adverse events
SAS: Safety Analysis Set
