# Supplementary material for: Antibiotic therapy in viral airway infections (ATHENIAN): study protocol for an open labeled randomized controlled pragmatic trial to evaluate the efficacy and safety of discontinuing antibiotic therapy in adult patients infected with respiratory viruses
Source: Trials. 2026 Apr 21;27:410. doi: 10.1186/s13063-026-09730-3 (PMC13231704; doi:10.1186/s13063-026-09730-3)
Supplement: Supplementary file 1 — Supplementary Material 1. [file 13063_2026_9730_MOESM1_ESM.pdf]

| | |
|---|---|
| Sponsor name | **Akershus University Hospital**<br>Jan Erik Berdal |
| Sponsor address | **Akershus University Hospital**<br>Head of Department / Associate Professor Jan Erik Berdal<br>Department of Infectious Diseases<br>Akershus University Hospital<br>Sykehusveien 25<br>1478 Lørenskog, Norway<br>Tel: 67 96 00 00<br>E-mail: jan.erik.berdal@medisin.uio.no |
| EudraCT number | 2021-004248-11 |
| Trial ID | Ahus-ATHENIAN |

# Antibiotic THErapy iN vIral Airway iNfections (ATHENIAN): An open labelled randomized controlled pragmatic trial to evaluate the efficacy and safety of discontinuing antibiotic therapy in adult patients infected with respiratory viruses

Statistical Analysis Plan for the final analysis

Version 1.5

Date: 18MAR2026

## SIGNATURE PAGE

**SPONSOR REPRESENTATIVE:**

**Head of Department / Associate Professor Jan Erik Berdal, MD PhD**
Department of Infectious Diseases, Division of Medicine
Akershus University Hospital

Signature

Date (dd/mmm/yyyy)

**PRINCIPAL INVESTIGATOR:**

**Professor Magnus Nakrem Lyngbakken, MD PhD**
Department of Infectious Diseases, Division of Medicine
Akershus University Hospital

Signature

Date (dd/mmm/yyyy)

**TRIAL STATISTICIANS:**

**Jurate Saltyte Benth, PhD**
Health Services Research Unit
Akershus University Hospital

Signature

Date (dd/mmm/yyyy)

**ABBREVIATIONS**

| | |
|---|---|
| AE | Adverse Event |
| ATC | Anatomical/ Therapeutic/ Chemical |
| BMI | Body Mass Index |
| CI | Confidence Interval |
| DMC | Data Monitoring Committee |
| eCRF | electronic Case Report Form |
| EDC | Electronic Data Capture |
| ICU | Intensive Care Unit |
| ID | Identification/ Identifier |
| IMP | Investigational Medicinal Product |
| ITT | Intention to treat |
| MedDRA | Medical Dictionary for Regulatory Activities |
| NEWS | National Early Warning Score |
| PCT | Pragmatic Clinical Trial |
| PP | Per-protocol |
| RCT | Randomised Controlled Trial |
| SAE | Serious Adverse Event |
| SAP | Statistical Analysis Plan |
| SD | Standard Deviation |
| SPC | Summary of Product Characteristics |
| SUSAR | Suspected Unexpected Serious Adverse Reactions |
| WHO | World Health Organization |

## TABLE OF CONTENTS


1 STUDY OBJECTIVES ..... 5
1.1 Primary Objective ..... 5
1.2 Secondary Objectives ..... 5
2 OVERALL STUDY DESIGN ..... 5
3 GENERAL STATISTICAL CONSIDERATIONS ..... 6
4 HYPOTHESES AND DECISION RULES ..... 7
4.1 Statistical Hypotheses ..... 7
5 DEFINITIONS AND DERIVED VARIABLES ..... 8
5.1 Calculations ..... 8
5.2 Safety definitions ..... 8
6 EFFICACY AND SAFETY ENDPOINTS / VARIABLES ..... 8
6.1 Primary Endpoint ..... 8
6.2 Secondary Endpoints ..... 8
6.3 Safety Parameters ..... 9
7 DETERMINATION OF SAMPLE SIZE ..... 9
7.1 Randomization procedure ..... 10
8 DATA SETS TO BE ANALYSED ..... 10
8.1 Protocol violations ..... 10
9 BLIND REVIEW ..... 11
10 STATISTICAL METHODOLOGY ..... 11
10.1 Adjustment for covariates ..... 11
10.2 Centre effect ..... 11
10.3 Multiplicity adjustments ..... 11
10.4 Demographic and other baseline characteristics ..... 11
10.5 Primary analysis ..... 11
10.6 Secondary analyses ..... 12
10.7 Subgroup analyses ..... 12
10.8 Patient Characteristics ..... 12
10.9 Exploratory Analysis ..... 13
11 SAFETY ANALYSIS ..... 13
11.1 Adverse events ..... 13
11.2 Other safety assessments ..... 13
12 INTERIM ANALYSIS ..... 13
13 DATA MONITORING ..... 14
14 TABLE OF CONTENTS OF TABLES AND FIGURES ..... 14
14.1 List of planned Tables ..... 14
14.2 List of planned Figures ..... 14
15 REFERENCES ..... 14
16 APPENDICES ..... 14

## AMENDMENTS TO THE SAP

Not applicable

# 1 STUDY OBJECTIVES

## 1.1 Primary Objective

In patients with positive airway sample for respiratory viruses, assess whether discontinuation of antibiotic therapy is non-inferior to continuation of antibiotic therapy with regard to early clinical response. Early clinical response is quantified as survival with symptom improvement without receipt of rescue antibacterial therapy. Early clinical response is defined as improvement of one or more levels relative to baseline in two or more symptoms of the investigator's assessment of symptoms of community-acquired bacterial pneumonia and no worsening of one or more levels in other symptoms. If, on follow-up, it is not possible for two symptoms to improve by one or more levels relative to baseline, then symptom improvement will be defined as either an improvement of one or more levels in one symptom, or any improvement that results in a symptom reaching—or remaining at—'no symptoms'.

## 1.2 Secondary Objectives

In patients with positive airway sample for respiratory viruses, assess whether discontinuation of antibiotic therapy is comparable to continuation of antibiotic therapy with regard to

1. In-hospital mortality and 30-day mortality
2. Duration of hospital admission
3. Days of therapy with antibiotics
4. Rescue antibiotic therapy during hospital admission
5. Frequency of new antibiotic therapy up to 30 days after discharge
6. Hospital readmission up to 30 days after discharge

# 2 OVERALL STUDY DESIGN

The study is a two-arm, open label, pragmatic randomized controlled non-inferiority stop trial designed to assess the safety and efficacy of discontinuing antibiotic therapy in patients with positive airway sample for respiratory viruses. Pragmatic clinical trials (PCT) are characterized by 3 attributes: (1) focus on informing decision-makers (e.g. patients, politicians, administrators) on optimal clinical medicine practice, as opposed to elucidating a biological or social process; (2) intent to enrol a population representative to the decision in practice and for whom the decision is relevant; and (3) either an intent to streamline procedures and data collection in the trial or to measure a broad range of outcomes. By utilizing resources already paid for by the hospitals (physicians and nurses in daily clinical practice), PCT can include a larger number of patients at a short time duration and at a lower cost than studies utilizing traditional randomized controlled trial (RCT) designs with an external study organization (e.g. study nurses, study physicians). A pragmatic approach will enable swift initiation of randomization and treatment. We will especially use data from the data warehouse solutions at the trials centres for eligible patient identification (i.e. electronic surveillance) and for automatic data extraction to the study specific database. The study will examine the effects of discontinuing treatment and will accordingly not be placebo-controlled.

In the initial phase of the study, patients will be included from a single centre (Akershus University Hospital). The study will perform interim safety analyses after the inclusion of 40 patients, and subsequent interim analyses will be performed after 100 and 200 completed patients. Extraordinary interim meeting will also be conducted if deemed necessary by the Sponsor. This approach will enable frequent assessment of study safety measures. By the winter of 2023/2024 and after additional external funding for the study, the study will expand to a multicentre study with multiple sites in Norway. See Section 10 Statistical methods and data analysis for further details.

All patients at including hospitals with suspicion of acute respiratory tract infections are examined with a nasopharyngeal swab, with subsequent microbiological examination, including specific polymerase chain reaction (PCR) for respiratory viruses. Laboratory reports from the Department of Microbiology are surveilled in real-time for positive samples, which will allow for immediate screening and randomization in all eligible subjects. **Figure 1** gives a schematic overview of the study.

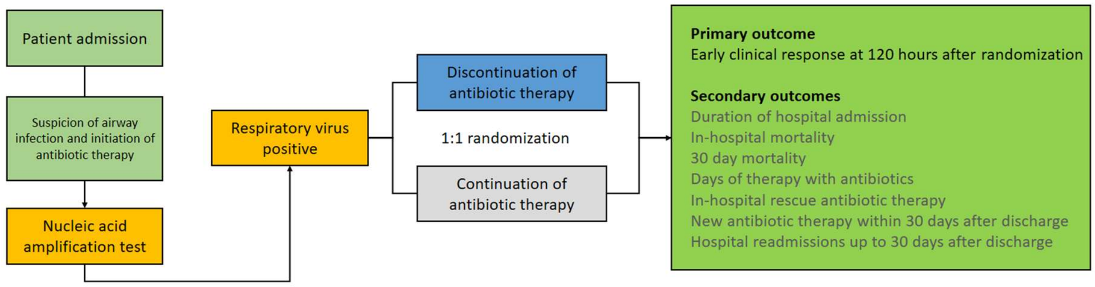


**Figure 1.** Trial design

| | |
|---|---|
| Study Period of initial study phase: | First patient enrolled: 13.01.22 |
| | Anticipated recruitment period: 72 months |
| | End of study (last patient's last visit): 01.02.28 |
| | Estimated study termination: 01.02.33 |
| | Time-frame of study data retention: 01.02.58 |
| Treatment Duration: | At the discretion of the treating physician in the control arm of the study. |
| Follow-up: | At five and 30 days. |

# 3 GENERAL STATISTICAL CONSIDERATIONS

All described analyses are considered *a priori* analyses in that they have been defined in the protocol and/or this SAP. All *post hoc* analyses will be identified as such in the Clinical Study Report. Even though this is an open trial, the SAP and choice of statistical programs were specified prior to any analyses, tabulations, or figures. Thus, for all practical purposes, the statistician writing this SAP can be considered blinded. In case of inconsistency between the SAP and the statistical chapter of the study protocol, the SAP will have precedence.

All categorical (binary and ordinal) data will be summarised using frequency counts and percentages. Percentages will be calculated using the study population; any exceptions to this will be highlighted in the table footnote. The continuous variables will be summarised using number of patients (N), mean, standard deviation (SD), median, 25$^{th}$/75$^{th}$ percentiles and minimum/maximum values, depending on the distribution. In general, minimum and maximum will be presented to the same degree of precision as data is recorded, with mean and median rounded to the nearest tenths and SD rounded to the nearest hundredths. Percentages less than 100 will be displayed with tenths.

Effect estimates will be reported together with 95% confidence intervals (CIs) and p-values. P-values will be rounded to the nearest thousandths. P-values below 0.001 will be displayed as "<0.001". Data will be presented, unless otherwise indicated, stratified by the two intervention groups.

In general, results presented in the tables and figures will be confined to those subjects included in the safety analysis set, full analysis set and per protocol set (see below), or a specific subset thereof.

Study days will be computed from the date of randomization, with study day 1 representing the first treatment date.

If the amount of missing follow-up (120 hours after randomization) data for the primary endpoint is considered likely to meaningfully influence the trial conclusions, a sensitivity analysis will be conducted using inverse probability weighting to assess the robustness of the results. The weights will be generated by using relevant baseline patient characteristics in order to assure as balanced groups as possible. This approach aims to mitigate potential bias arising from informative missingness. The same strategy will be applied to secondary endpoints if similar concerns regarding missing data arise.

This trial is designed for 3 interim analyses and one final analysis. The interim analyses will be performed for the safety endpoints, and the final analysis for all endpoints described in the SAP. The interim analyses will be performed by a designated Data Monitoring Committee (DMC) statistician, who will be unblinded to treatment allocation.

# 4 HYPOTHESES AND DECISION RULES

## 4.1 Statistical Hypotheses

This study is primarily designed to establish the non-inferiority with regard to early clinical response of discontinuing of antibiotic therapy in patients with positive airway sample for respiratory viruses.

The null hypothesis is that discontinuation of antibiotic therapy in patients with positive airway sample for respiratory viruses is inferior to continuing antibiotic therapy with regard to early clinical response.

The alternative hypothesis is that discontinuation of antibiotic therapy in patients with positive airway sample for respiratory viruses is non-inferior to continuing antibiotic therapy with regard to early clinical response.

The null hypothesis will be tested using an appropriate statistical test as described in chapter 10.5.

# 5 DEFINITIONS AND DERIVED VARIABLES

For analysis and tabulation purposes, we define the study time points (TPs) as follows:

| Time Point Label | Target Hour/Day | Definition (Hour/Day window) |
|---|---|---|
| TP1. Baseline | Hour 0 (Randomization) | 0 hours |
| TP2. 120 hours | Hour 120 | 96-144 hours |
| TP3. 30 days | Day 30 | 29-31 days |

## 5.1 Calculations

Age (years) = [(date of baseline – date of birth)/365.25].

Time of withdrawal = (date of withdrawal – date of randomization)

Follow-up time = (last date in study – date of randomization)

In-hospital time = (discharge date – admission date)

BMI = weight in kilograms / (height in metres x height in metres)

BMI will be categorized according to the WHO definitions for underweight, normal, overweight and obese: <18.5 kg/m2 (underweight), 18.5–24.9 kg/m2 (normal range), 25-29.9 (overweight), ≥30 (obesity)

## 5.2 Safety definitions

### 5.2.1 Treatment emerging adverse events

Treatment emerging adverse events (TEAEs) are defined as AEs with a start date on or after the first treatment day. As described in the main study protocol, only SAE and SUSAR will be registered in the trial.

# 6 EFFICACY AND SAFETY ENDPOINTS / VARIABLES

## 6.1 Primary Endpoint

The primary endpoint is early clinical response defined as proportion surviving with symptom improvement without receipt of rescue antibacterial therapy. Symptom improvement is defined as improvement of one or more levels relative to baseline in two or more symptoms of the investigator's assessment of symptoms of community-acquired bacterial pneumonia and no worsening in any other symptoms. If, on follow-up, it is not possible for two symptoms to improve by one or more levels relative to baseline, then symptom improvement will be defined as either an improvement of one or more levels in one symptom, or any improvement that results in a symptom reaching—or remaining at—'no symptoms'. See also chapter 10.5 on the primary analysis. The primary endpoint will not be assessed in the interim analyses.

## 6.2 Secondary Endpoints

The secondary endpoints will not be assessed in the interim analyses.

### 6.2.1 Efficacy

Following efficacy endpoints will be assessed:

In-hospital mortality and 30-day mortality

Duration of hospital admission

Days of therapy with antibiotics

Rescue antibiotic therapy during hospital admission

New antibiotic therapy up to 30 days after discharge

Hospital readmission up to 30 days after discharge

### 6.2.2 Patient Reported Outcomes (PRO)

None

## 6.3 Safety Parameters

For each patient, the collection and recording SAEs will begin at the day of randomization and will continue till 120 hours after randomization. In the case of patient's discharge before the end of follow-up, patient will be followed up by phone for assessment of the primary end point and SAEs. AEs will not be reported unless they meet the definition of SAEs.

- Adverse events (AEs): any unfavourable and unintended sign (including an abnormal laboratory finding), symptom, or disease temporally associated with the use of a medicinal (investigational) product, whether or not related to the medicinal (investigational) product. The term AE is used to include both serious and non-serious AEs.

- Serious adverse events (SAEs): any untoward medical occurrence that at any dose 1) Results in death; 2) Is immediately life-threatening; 3) Requires in-patient hospitalization or prolongation of existing hospitalization; 4) Results in persistent or significant disability or incapacity; 5) Is a congenital abnormality or birth defect; or 6) Is an important medical event that may jeopardize the subject or may require medical intervention to prevent one of the outcomes listed above.

- Suspected unexpected serious adverse reactions (SUSARs): an SAE that is unexpected as defined after and possibly related to the Investigational Medicinal Product (IMP). Any event other than those mentioned in the Summary of Product Characteristics (SPC) of the IMP will be classified as SUSAR.

# 7 DETERMINATION OF SAMPLE SIZE

The clinical course of viral airway infections is heterogeneous and depends on patient demographics and comorbidities, as well as causative viral agent. In patients with influenza, clinical improvement is expected at around day 3 to 7. The proportion of study patients with early clinical improvement from randomization to 120 hours will be used as the basis for determination of sample size. Early clinical improvement does not equate to complete resolution of symptoms, and the in-hospital mortality rate of moderately ill patients admitted with viral airway infections is very low.

Accordingly, we assume an early clinical improvement of 90% in both treatment groups, with a non-inferiority margin of 10%. Under these assumptions using a 2.5% one-sided significance level and 90% power, we would need to include a total of 380 patients (190 randomized to the intervention group and 190 randomized to the control group). To account for screening failures and loss to follow-up, we aim to include up to 400 patients in the current study. The first interim analysis will be performed after 40 included patients, and subsequent interim analyses will be performed after 100 and 200 included patients. The study will be conducted at multiple sites in Norway and a possible clustering effect on site level might be present. However, Norway has a universal health care system with equal access for all citizens and patient treatment algorithms for hospital admitted patients are homogenous throughout the country. There is therefore little reason to believe that treatment and short-term outcomes for respiratory tract infections are vastly different in Norwegian hospitals, and the study intervention is expected to have similar effect across including sites, which are predominantly University hospitals and large regional hospitals. Due to a negligible risk of cluster effect, there was no adjustment implemented in the power calculations.

## 7.1 Randomization procedure

Eligible patients will be allocated in a 1:1 ratio, using a computer-generated block-randomization procedure. The allocation sequence will be prepared by an independent statistician. The randomization sequence will use block randomization with randomized block sizes (2, 4, 6 and 8), and a separate randomization sequence will be prepared for each study site. At every newly included site, the stratified randomization will be performed to assure balanced arms within the site. No such stratification is performed in Akershus University Hospital.

# 8 DATA SETS TO BE ANALYSED

The **Full Analysis Set (FAS)** will include all randomised subjects who have had at least one baseline and one post-randomisation evaluation for the primary end point.

The **Intention-to-Treat (ITT)** population will include all randomized participants, analysed according to their assigned treatment groups regardless of treatment adherence, protocol deviations, or availability of post-baseline data.

**The per-protocol (PP) set** is a subset of the FAS including all randomised participants without major protocol violations.

The **Safety Analysis Set (SAS)** includes all subjects with any safety information after baseline.

The analysis of primary endpoint will be performed on the FAS (corresponding to modified intention-to-treat, mITT) and the PP set *(1)*. The analysis of the secondary endpoints will be performed on the ITT and PP set. The safety analysis will be performed on the SAS.

## 8.1 Protocol violations

The following criteria need to be fulfilled for participants to be included into the PP set:

- No violation of eligibility criteria
- Full data set allowing analysis of the study primary endpoint

# 9 BLIND REVIEW

Prior to statistical analyses, the data in the data base will be exported and the exported data will be closed or changes. The study statistician will be blinded to the randomization allocation until all statistical analyses are performed. The statistical interim analysis will be performed by an unblinded Data Monitoring Committee's (DMC) statistician based on program code from the trial statistician. The unblinded statistician performing the interim analyses will only provide the study group with information on whether the trial should be stopped or continued.

# 10 STATISTICAL METHODOLOGY

All results will be presented as the size (point estimate) of the difference between the treatments with the corresponding 95% CIs and p-values.

## 10.1 Adjustment for covariates

There will be no adjustment for baseline or other covariates.

## 10.2 Centre effect

The centre effect is expected to be negligible. Thus, no adjustment in the power calculations and planned statistical analyses will be made.

## 10.3 Multiplicity adjustments

As there is only one pre-defined primary analysis, there will be no adjustment for multiple testing implemented.

## 10.4 Demographic and other baseline characteristics

Demographic and baseline characteristics will be summarised descriptively by treatment group using the FAS. Categorical data will be displayed as frequencies and percentages. Continuous data will be displayed as means, SDs, and minimum/maximum values, or in case of skewed distribution, as medians and 25$^{th}$ and 75$^{th}$ percentiles. The analyses will include baseline characteristics such as symptoms, imaging, co-morbidities, substance use, clinical biochemistry, microbiology, anthropometrics, NEWS score, current medications, etc.

## 10.5 Primary analysis

The primary outcome is early clinical response defined as a combination of all of the following:

1. Survival
2. Improvement of one or more levels relative to baseline in two or more symptoms of the investigator's assessment of symptoms of community-acquired bacterial pneumonia and no worsening in any other symptoms. If, on follow-up, it is not possible for two symptoms to improve by one or more levels relative to baseline, then symptom improvement will be defined as either an improvement of one or more levels in one symptom, or any improvement that results in a symptom reaching—or remaining at—'no symptoms'.
3. No rescue antibiotic therapy

The primary endpoint will be analysed as the difference in proportions between the intervention and control groups. The confidence interval (CI) for the difference will be calculated using the standard normal approximation (Wald metho). A one-sided 97.5% CI will be presented. Non-inferiority will be

concluded if the lower bound if the 97.5% CI exceeds the prespecified non-inferiority margin (Chapter 7). If non-inferiority is demonstrated, superiority in primary outcome will be assessed.

As a sensitivity analysis, the effect of the intervention on a version of the primary outcome where criterion 3 (i.e., no rescue antibiotic therapy) is removed will be assessed. Employing this approach will increase robustness of the primary inference and generalizability of the results to health care systems with different antibiotic treatment policies. For the sensitivity analysis, the same non-inferiority margin and confidence interval will be used for assessing the non-inferiority hypothesis.

## 10.6 Secondary analyses

The between-group comparisons for secondary endpoints will be tested based on the following methods (but not limited to):

- Continuous variables will be subject to Independent-samples t-test or appropriate non-parametric alternatives.
- Time-to-event endpoints and durations will be assessed by the Kaplan-Meier curves and compared by the log-rank test.
- Binary and categorical response variables will be compared by $\chi^2$-test.

## 10.7 Subgroup analyses

The primary and secondary analysis results will be assessed separately according to the following subgroups:

- Sex
- Age above/below 65 years
- Admission C-reactive protein above/below 100 mg/L
- Patients with/without history of diabetes mellitus
- Patients with/without history of obstructive pulmonary disease (COPD or asthma)
- Patients with/without history of cardiovascular disease
- Type of respiratory virus detected (influenza, parainfluenza virus, RSV, or hMPV)
- Patients with and without radiographic evidence of pneumonia (lobar infiltrates, bronchopneumonia, etc)

## 10.8 Patient Characteristics

### 10.8.1 Patient Disposition

The disposition of all patients will be listed and summarised by treatment arm. The number and percentage of patients who are randomised, received any study treatment, prematurely discontinued from treatment, and lost to follow-up will be summarised.

The number and percentage of patients will be categorized by the reason(s) of loss to follow-up. i.e., when the patient withdraws or is withdrawn from the study. Reasons can be: violation of eligibility criteria, patient withdrawal of informed consent, adverse event, death, investigator decision or other.

### 10.8.2 Protocol Deviations

Protocol deviations resulting in exclusion from the PP set will be determined and summarised by treatment group. See section 8.2 for protocol deviation categories.

#### 10.8.3 Background and Demographic Characteristics

Patient demographics and baseline characteristics will be summarised for the FAS.

Patient demographics and baseline characteristics will be summarised by randomised treatment arm using descriptive statistics (N, means, SDs, medians, $25^{th}/75^{th}$ percentiles, minimum, and maximum) for continuous variables, and frequencies and percentages of patients for categorical variables.

#### 10.8.4 Concomitant Medications and Other Therapies

Concomitant medication information will be coded by the Anatomical Therapeutic Chemical (ATC) classification system. Concomitant medications taken during the study will be summarised by generic name. Patients will only be counted once if they are taking more than one medication (within the same code) or take the same generic medication more than once.  If it cannot be determined whether a medication is concomitant (based on stop date or, if the stop date is missing, start date), then the medication will be considered to be concomitant. Information on concomitant medication will be extracted from the hospital electronic journal system.

### 10.9 Exploratory Analysis

Exploratory analyses will be defined at a later time point and will not be performed or are specified at the time point of the first interim analysis. Later versions of this SAP will detail the exploratory analyses.

Exploratory endpoints will not be limited to those mentioned here and will include variables/endpoints and statistical methods/modelling as necessary to explore the secondary objectives of the study as described in the protocol.

## 11 Safety Analysis

Safety analysis will be based on the safety analysis set. Safety analyses will be descriptive and presented as summary tables by treatment group.

### 11.1 Adverse events

We will calculate the numbers and proportions of participants in the safety analysis set that experienced an SAE, and SUSAR by study arm for the time interval from baseline to 120 hours post-randomisation. We will also present the number and proportion of participants with 1, 2, or ≥3 SAEs, and SUSARs; the number and proportion of participants with any treatment-related SAE. Further, we will display the total number of SAEs, and SUSARs, independent of number of participants. No formal statistical testing of differences between the study arms will be performed.

All deaths and SAEs recorded during this trial will be reported.

### 11.2 Other safety assessments

No further safety parameters will be assessed or reported.

## 12 Interim analysis

This trial is designed to perform three interim analyses and will include safety analysis supporting continuation or discontinuation of the study. Details about the statistical analyses and decision rules for the interim analyses are provided in the DMC chapter.

# 13 Data monitoring

Details on data monitoring, Data Monitoring Committee (DMC) and interim analyses are provided in the DMC chapter.

The DMC will meet three times after 40, 100 and 200 patients have completed the assessment of the primary endpoint to review unblinded safety data. Stopping and decision rules are defined in the DMC chapter.

# 14 Table of Contents of Tables and Figures

The study group will receive a mock report (complete report but with a dummy study group allocation) from the trial statistician for each of the interim and the final analysis. Based on this report, the study group will find consensus on the final tables and figures to be reported.

## 14.1 List of planned Tables

Table 1: Patient disposition

Table 2: Demographic and baseline characteristics by study arm (only age and sex for interim analysis)

Table 3: Results of the analysis of the primary endpoint (not relevant for interim analyses)

Table 4: Results of the analysis of the secondary endpoints (not relevant for interim analyses)

Table 5: Safety tables (SAEs, SUSARs)

Table 6: Protocol deviations and treatment adherence (not relevant for the interim analyses)

## 14.2 List of planned Figures

Figure 1: Summary of patient enrolment

# 15 References

1. Bai AD, et al. Intention-to-treat analysis may be more conservative than per protocol analysis in antibiotic non-inferiority trials: A systematic review. BMC Medical Research Methodology 2021;21:75.

# 16 Appendices
